# Lipid accumulation product and visceral adiposity index are associated with dietary patterns in adult Americans

**DOI:** 10.1097/MD.0000000000010322

**Published:** 2018-05-11

**Authors:** Mohsen Mazidi, Hong-kai Gao, Andre Pascal Kengne

**Affiliations:** a Key State Laboratory of Molecular Developmental Biology, Institute of Genetics and Developmental Biology, Chinese Academy of Sciences; b Department of General Surgery, The General Hospital of Chinese People's Armed Police Forces, Beijing, China; c Non-Communicable Disease Research Unit, South African Medical Research Council and University of Cape Town, Cape Town, South Africa.

**Keywords:** Glucose, insulin, lipid accumulation product, visceral adiposity index

## Abstract

In the present study, we aimed to examine the association between lipid accumulation product (LAP) and visceral adiposity index (VAI) with dietary pattern (DP) in the US adults. Participants of the National Health and Nutrition Examination Survey (NHANES) with data available on dietary intake from 2005 to 2010 were included. DPs were derived by principal component analysis. We applied analysis of covariance and multivariable-adjusted linear regressions accounting for the masked variance and utilizing the proposed weighting methodology. The analytical sample comprised 18,318 participants (mean age = 45.8 years), of whom 48.3% (n = 8607) were men with no age difference by gender (*P* = .126). The first DP was representative of a diet rich in carbohydrate and sugar, total fat and saturated fatty acid (SFA), high-caloric dieatry pattern; the second DP was highly loaded with vitamins, minerals and fiber (nutrient-dense dietary patten), and the third DP was mainly representative of high dietary polyunsaturated fatty acids (PUFAs) and monounsaturated fatty acids (MUFAs) (healthy fat DP). The adjusted (age, sex, race, physical activity, smoking, C-reactive protein) mean of LAP, VAI and glucose homeostasis indices increased across increasing quarters of the first DP score (all *P* < .001), while across increasing score of the second DP, the adjusted mean of LAP, VAI, glucose homeostasis indices decreased (all *P* < .001). Findings were similar in adjusted linear regressions models. Our findings support that affordable measurements, such as VAI and LAP, could be good alternative surrogate markers of visceral fat. They are also significantly related to DPs in same line as with glucose/insulin homeostasis and anthropometric indices.

## 1 Introduction

Excess visceral adipose tissue (VAT) is one of the most deleterious fat depots in the body, with strong links with cardiovascular disease, and certain types of cancer.^[1,2]^ Lipid accumulation product (LAP) index, a recently developed biomarker of central fat accumulation, has been recommended as a precise indicator of the risk of insulin resistance, metabolic syndrome, type 2 diabetes, and cardiovascular disease.^[3–5]^ Higher LAP has been associated with abnormal glucose homeostasis and insulin resistance, as well as elevated alanine aminotransferase in healthy individuals.^[6]^ A Chinese study has shown that both LAP and visceral adiposity index (VAI) were effective markers for stratifying adults for obesity phenotypes.^[7]^ In addition, another study reported that LAP was a helpful indicator for the screening for metabolic syndrome.^[8]^

The VAT seems to be affected by diet and lifestyle modifications.^[9,10]^ Furthermore, it has been suggested that VAT is mainly influenced by the non-caloric qualitative aspects of diet, although evidence on the association between macronutrient composition of diet and VAT, is still limited. A recent investigation indicated that consuming energy mainly as carbohydrate or fat for 3 months did not affect visceral fat and metabolic syndrome in a low-processed, lower-glycemic dietary context.^[11]^ There are contradictory findings regarding the association between different dietary patterns (DPs), LAP, and VAI. Significant association between carbohydrate intake,^[12,13]^ dietary fatty acids^[14]^ including saturated fatty acids (SFAs), monounsaturated fatty acids (MUFAs), polyunsaturated fatty acids (PUFAs) with VAT has not been reported in all investigations.^[8]^ However, it is important to note that foods and nutrients are consumed in combination, and complex combinations of nutrients are likely to be interactive or to have a synergistic effect.^[15]^ The approach of evaluating single nutrients or foods might therefore be limited in terms of potential interactions and high inter-correlations between several food components, which might make it challenging to estimate the general, or independent impacts of different nutrients or foods, perhaps slight and thus untraceable impacts of a single nutrient may be concealed, and the concern of multiple comparison is also crucial in this area.^[16]^ Therefore, in an efforts to overcome these issues, the analysis of DPs has gained importance.^[15,17]^ A DP is a comprehensive variable that incorporates the intake of numerous nutrients or nutrient groups and that has a more impact on disease risk than does any single nutrient.^[16,17]^

The mechanisms by which nutrient patterns affect the risk of chronic conditions are not fully understood and there is good evidence that it is a combination of nutrients, rather than an individual one, that will affect the risk. Therefore, a pattern of nutrients may provide more information about probable underlying mechanisms.^[18,19]^

The aim of present study is to investigate the association between LAP, VAI with DPs, alongside markers of glucose/insulin homeostasis (which are well-characterized correlates of VAT) in randomly selected nationally representative samples of the US adults.

## 2 Methods

### 2.1 Population

The National Health and Nutrition Examination Surveys (NHANESs) conducted between 2005 and 2010 were used for this study. NHANESs are repeated cross-sectional surveys conducted by the US National Center for Health Statistics, applying protocols and procedures described in details previously.^[20,21]^ NHANES uses a complex, multistage, and stratified sampling design to select a sample representative of the civilian and non-institutionalized resident population of the United States. Data on demographic information and interviews are collected using questionnaires administered during home visits, while anthropometrical, inflammation, and biochemistry data are collected by trained personnel using mobile examination units. Methods for biochemical analyses are described in the NHANES Laboratory/Medical Technologists Procedures Manual.^[20,22–24]^ NHANES is open access database and all the information on the data access and analysis can be found at https://www.cdc.gov/nchs/nhanes/index.htm.

Dietary intake was assessed via 24 hours recall obtained by a trained interviewer during the mobile examination center visit, with the use of a computer-assisted dietary interview system with standardized probes, that is, the United States Department of Agriculture Automated Multiple-Pass Method (AMPM).^[25,26]^ Briefly, the type and quantity of all foods and beverages consumed in a single 24-hour period before the dietary interview (from midnight to midnight) were collected with the use of AMPM. AMPM is designed to enhance complete and accurate data collection while reducing respondent burden.^[26,27]^

A blood specimen was drawn from the participant's antecubital vein by a trained phlebotomist. Glycated hemoglobin (HbA1c) was measured using a Tosoh A1C 2.2 plus glycohemoglobin analyzer (San Francisco). Fasting plasma glucose was measured by a hexokinase method using a Roche/Hitachi 911 analyzer (New Jersey) and Roche Modular P chemistry analyzer (New Jersey). Insulin was measured using an enzyme-linked immunosorbent assay (Merocodia, Uppsala, Sweden).^[20]^ Homeostatic model assessment of insulin resistance (HOMA-IR) was calculated as follows: (FBG [nmol/L] × Insulin [mU/mL]/22.5) using fasting values.^[28]^ Other laboratory-test details are available in the NHANES Laboratory/Medical Technologists Procedures Manual.^[29]^ Details on information on high-sensitivity C-reactive protein (hsCRP) concentration measurements are available elsewhere.^[24]^

The triglyceride (TG)-glucose (TyG) index was calculated as the ln(Fasting TG [mg/dL] × Glucose [mg/dL]/2).^[30]^ The anthropometrically predicted VAT (apVAT) was estimated with sex-specific validated equations that included age, body mass index (BMI), and circumferences of the waist (WC) and thigh.^[31]^ The equation for men was: 6 × WC – 4.41 × Proximal thigh circumference + 1.19 × Age – 213.65; and the equation for women was: 2.15 × WC – 3.63 × Proximal thigh + 1.46 × Age + 6:22 × BMI − 92.713.^[31]^ VAI was calculated using sex-specific formulas: males (WC/39.68 + [1.88 × BMI]) × (TGs/1.03) × (1.31/high-density lipoprotein [HDL]); females: (WC/36.58 + [1.89 × BMI]) × (TGs/0.81) × (1.52/HDL), where both TGs and HDL levels are expressed in mmol/L.^[32]^ LPA was calculated as (WC/65) × TG in men, and (WC/58) × TG in women.^[33]^

### 2.2 Statistical analysis

We analyzed the data in compliance with the prescribed guidelines for analysis of complex NHANES data set, taking into account the masked variance and utilizing the proposed weighting methodology.^[20,34]^ Factor analysis was applied with orthogonal transformation (varimax procedure) to derive DPs based on the nutrients and bioactive compounds. Factors were retained for further analysis based on their natural interpretation and eigenvalues on the Screen test.^[35]^ We computed the factor score for each nutrient pattern by summing up intakes of nutrients weighted by their factor loadings.^[35]^ Each participant received a factor score for the identified pattern. We categorized the subjects based on quarters of nutrient pattern scores. We computed age, sex, race, physical activity, smoking, CRP, and history of diabetese adjusted means of our outcomes across quarter of DPs by using analysis of covariance. Adjusted multivariate linear regressions (age, sex, race, physical activity, smoking, CRP) were used to examine the association of score of food pattern with adiposity. All tests were two-sided, and *P* < .05 was the level of significance.

## 3 Results

The analytical sample comprised 18,318 participants, of whom 48.3% (n = 8607) were men. The mean age was 45.8 years in the overall sample and did not vary significantly in men and women (*P* = .126). White (non-Hispanic) participants formed the majority (69.4%) of the population. Furthermore, 56.1% (n = 8759) of the participants were married, and 19.8% were current smokers (23.9% of men and 16.7% of women). The PCA method uncovered 3 DPs altogether explaining 55.9% of the variance in dietary nutrient consumption. The first DP was representative of a diet high in carbohydrate and sugar, total fat and saturated fatty acid (SFA), high-caloric dieatry pattern; the second DP was highly loaded with vitamins, minerals, and fiber (nutrient-dense dietary patten); and the third DP was mainly representative of high dietary PUFAs and MUFAs (healthy fat DP) (Table 1).

**Table 1:**
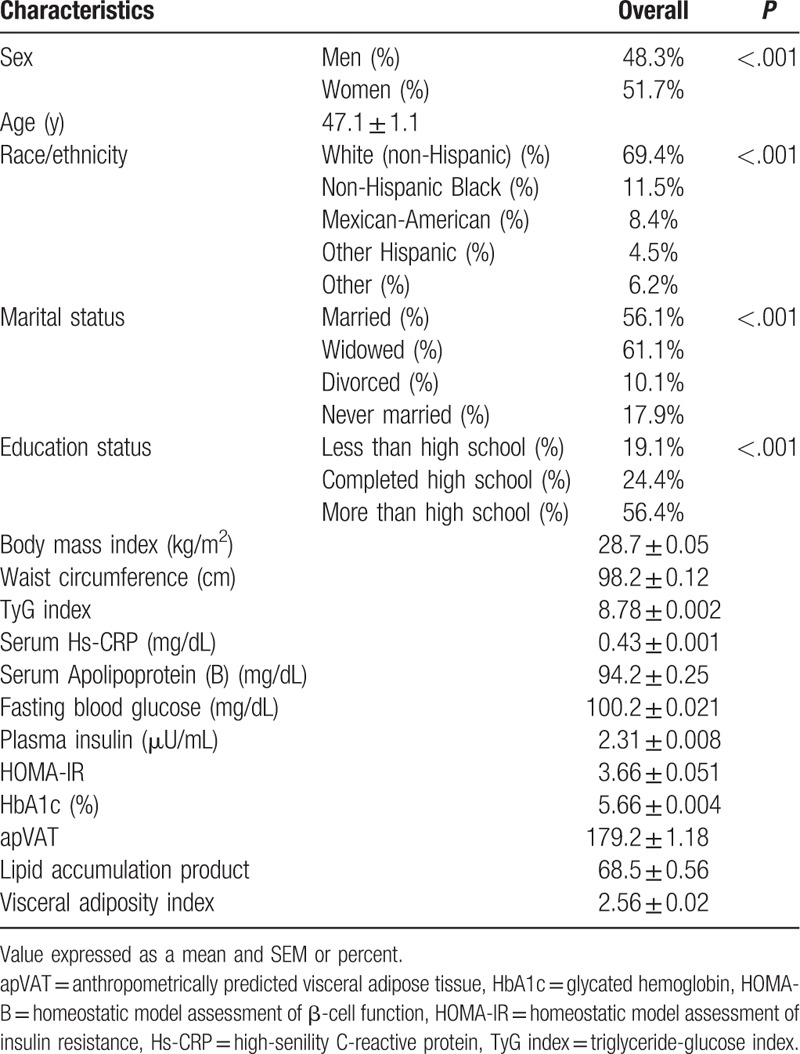
Demographic and clinical characters of subjects.

The adjusted (age, sex, race, physical activity, smoking, CRP) means of adposity factors (apVAT, LAP, VAI) and glucose/insulin homeostasis (FBG, insulin, HOMA-IR) increased across increasing quarters of the first DP score (all *P* < .001, Table 2), while across increasing score of the second DP, the adjusted mean of apVAT, LAP, VAI, FBG, insulin, and HOMA-IR decreased (all *P* < .001, Table 2). Across increasing quarters of the third DP, just LAP and FBG showed significantly decreasing trend (*P* < .001, Table 2). Adjusted linear regressions revealed a significant and positive association between first DP and adiposity and glucose/insulin homeostasis factors (all *P* < .001, Table 2), whereas there was a significant and negative association between second DP and same factors (all *P* < .001, Table 2).

**Table 2:**
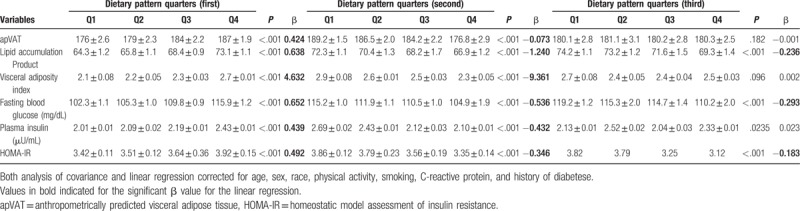
Adjusted mean of adiposity factors across quarters of dietary patterns and adjusted linear regression between adiposity factors and score of dietary patterns.

## 4 Discussion

Findings from this study revealed that adiposity factors and markers of glucose/insulin homeostasis were positively associated with the diet which highly consisting of the carbohydrate and sugar, total fat and SFA, and inversly associated with diet comprising vitamins, minerals, and fiber. Moreover, we found a negative association between LAP and diet highly loaded with PUFA and MUFA.

In contrast to our findings, in a prospective study, no relation was detected between SFAs, MUFAs, PUFAs, and 5-year percent change in VAT^[14]^; however, one cross-sectional study revealed a positive association between fat intake and VAT in overweight young adults aged 17 to 35 years.^[14]^ An Iranian investigation reported that increasing MUFA by decreasing total protein or PUFA in isoenergetic diets was positively associated with visceral adiposity index changes.^[36,37]^ The hypothesis that MUFAs are healthy fatty acids comes from studies investigating the impacts of olive oil, whereas further studies suggest MUFA intakes from animal sources to have different effects.^[36,37]^

Contrary to our results, some observational studies did not find a significant association between carbohydrate intake and VAT^[12,13]^; however, it has been proposed that replacing carbohydrate with total protein was positively associated with VAI in women only.^[37]^ A recent Iranian investigation reported that higher dietary proportions of protein and animal-derived MUFA could be positively associated with VAI; in addition, in isoenergetic diet, replacing carbohydrate, MUFAs, and PUFAs with protein was positively associated with 3-year changes in VAI.^[37]^ However, no significant association was reported between 2-year changes in total protein intake and change in VAT in 85 overweight adolescents aged 11 to 17 years,^[38]^ as well total protein intake was also not associated with 5-year percent change in VAT in 1114 black and hispanic overweight adults in another prospective study.^[14]^

An investigation reported that LAP and VAI were markers of insulin resistance and metabolic-related disturbances in young women with polycystic ovary syndrome.^[39]^ Recent meta-analysis investigated the effects of saturated fat, polyunsaturated fat, monounsaturated fat, and carbohydrate on glucose-insulin homeostasis.^[40]^ It reported that only energy intake substitution with PUFA was associated with lower fasting glucose, lower HbA1c, improved HOMA-IR, and improved insulin secretion capacity. Furthermore, insulin secretion capacity similarly improved when PUFA replaced MUFA. Experimental studies showed that PUFA suppresses oxidative stress, hepatic lipogenesis and steatosis, pancreatic lipotoxicity, and insulin resistance.^[41]^ In addition, MUFA consumption did not appear to significantly influence fasting glucose, compared to others macronutrients, however, was reported to reduce HbA1c and improve HOMA-IR in comparison to either carbohydrate or SFA.^[40]^

There are several limitations to this study. First, the results based on this cross-sectional study, although it is nationally representative, cannot demonstrate a causal relationship between DPs and VAT. Second, although our analysis included known potential confounding variables that can affect adiposity in terms of environmental and genetic factors, residual confounding variables may still exist. Moreover, we did not have data on the direct measurement of the VAT for validation. This study has several strengths. We had a large sample, selected randomly from general population; therefore, the results obtained from nationally representative samples can be extrapolated to the general population.

## 5 Conclusion

In conclusion, our findings suggest that LAP and VAI could potentially be used as indirect measures of VAT in routine setting and for research purpose, considering that they are likely more affordable than other advocated indirect measures such as markers of insulin resistance. Also they are significantly related to the DPs in same line with glucose/insulin homeostasis and anthropometrics indices.

## Author contributions

**Conceptualization:** Hong- Gao, Andre Pascal Pascal.

**Data curation:** Mohsen Mazidi.

**Formal analysis:** Andre Pascal Kengne, Mohsen Mazidi.

**Methodology:** Andre Pascal Pascal.

**Supervision:** Hong- Gao, Andre Pascal Pascal.

**Writing – original draft:** Mohsen Mazidi, Hong- Gao.

**Writing – review & editing:** Andre Pascal Pascal.
